# Nutrient Levels in Brassicaceae Microgreens Increase Under Tailored Light-Emitting Diode Spectra

**DOI:** 10.3389/fpls.2019.01475

**Published:** 2019-11-14

**Authors:** Giedre Samuolienė, Aušra Brazaitytė, Akvile Viršilė, Jurga Miliauskienė, Viktorija Vaštakaitė-Kairienė, Pavelas Duchovskis

**Affiliations:** Lithuanian Research Centre for Agriculture and Forestry, Institute of Horticulture, Babtai, Lithuania

**Keywords:** ascorbic acid, β-carotene, Brassicaceae, light-emitting diodes, microgreens, mineral elements, carbohydrates

## Abstract

To increase the nutritional value and levels of essential minerals in vegetable food, microgreens are promising targets. The metabolic processes of microgreens can be managed with different cultivation techniques, which include manipulating the properties of light derived by light-emitting diodes (LEDs). In this study *Brassicaceae* microgreens (kohlrabi *Brassica oleracea* var. *gongylodes*, broccoli *Brassica oleracea*, and mizuna *Brassica rapa* var. Japonica) were cultivated under different light spectral quality, and the metabolic changes insoluble sugars (hexoses and sucrose), ascorbic acid, β-carotene, and contents of non-heme iron (Fe) and its connection with magnesium (Mg) or calcium (Ca) levels were monitored. Plants grew under the primary LED light spectrum (the combination of blue light at 447 nm, red at 638 and 665 nm, and far-red at 731 nm) or supplemented with LED green light at 520 nm, yellow at 595 nm, or orange at 622 nm. The photoperiod was 16 h, and a total PPFD of 300 µmol m^-2^ s^-1^ was maintained. Under supplemental yellow light at 595 nm, the content of soluble carbohydrates increased significantly in mizuna and broccoli. Under all supplemental light components, β-carotene accumulated in mizuna, and ascorbic acid accumulated significantly in kohlrabi. Under supplemental orange light at 622 nm, Fe, Mg, and Ca contents increased significantly in all microgreens. The accumulation of Fe was highly dependent on promoters and inhibitors of Fe absorption, as demonstrated by the very strong positive correlations between Fe and Ca and between Fe and Mg in kohlrabi and broccoli, and the strong negative correlations between Fe and β-carotene and between Fe and soluble carbohydrates in kohlrabi. Thus, the metabolic changes that occurred in treated microgreens led to increases in the contents of essential nutrients. Therefore, selected supplemental LED wavelengths can be used in the cultivation of *Brassicaceae* microgreens to preserve and increase the contents of specific nutritionally valuable metabolites.

## Introduction

Microgreens are young, tender greens that are harvested at the first true leaf stage and sold with the stem, cotyledons (seed leaves), and first true leaves attached. Since the start of microgreen production in the late 1980s, the popularity of microgreen cultivation has continued to increase rapidly (Kyriacou et al., 2016). The seeds of almost any vegetable, herb, or grain, including those of wild species, can be used to grow microgreens. Microgreens have emerged in the market and have been popularized for their higher nutrient concentrations in the pair of first true leaves than those in their mature-leaf counterparts (Kyriacou et al., 2016; Xiao et al., 2019). According to Manchali et al. (2012), the most commonly consumed vegetables worldwide are those in the family *Brassicaceae*, including kohlrabi, broccoli, cabbage, cauliflower, radish, Brussels sprouts, or turnip. *Brassicaceae* vegetables are distinguished by high contents of glucosinolates, which are biologically active secondary metabolites involved in plant defense, flavor, taste, and human nutrition (Yang and Quiros, 2010). When increased in humans, the glucosinolates are hydrolyzed into several biologically active products, such as isothiocyanates and indoles, widely studied because of their antioxidant, anti-inflammatory and anticarcinogenic activity (Drewnowski and Gomez-Carneros, 2000; Baenas et al., 2017). The concentration and composition of glucosinolates vary significantly among different organs and developmental stages. The highest concentrations were found in reproductive organs and in young leaves (Brown et al., 2003).Yang and Quiros (2010) studied 82 different varieties of *Brassica rapa,* and although they did not find crop-specific glucosinolate, the predominant glucosinolates in most of the studied varieties were gluconapin, glucobrassicanapin (aliphatic), neoglucobrassicin, glucobrassicin (indolic), and gluconasturtiin (aromatic). Wagner et al. (2013) found that the primary breakdown product from predominant glucosinolates of broccoli is sulforaphane. In addition to glucosinolates, vegetables in the *Brassicaceae* accumulate high levels of antioxidant phytochemicals, such as ascorbic acid, carotenoids, phenolic compounds or tocopherols; thus they are favored for high antioxidant capacity (Domínguez-Perles et al., 2014; Xiao et al., 2015; Xiao et al., 2019). Some secondary metabolites are noted for their antiradical or antioxidant capability and play a significant role in regulating the oxidative damage caused by free radicals (Alrifai et al., 2019). Moreover, in an analysis of 30 varieties, Xiao et al. (2016) demonstrated that *Brassicaceae* microgreens are good sources of K and Ca as well as of Fe and Zn.

Ascorbic acid is an example of an important secondary metabolite (Pehlivan, 2017). Ascorbic acid is a cofactor for enzymes, is involved in regulating photosynthesis, has essential roles in biosynthesizing hormones, regulates cell division and growth, is involved in signal transduction, in addition to roles in detoxifying heavy metals, starting different radical reactions. Ascorbic acid can act as a prooxidant and increase iron absorption by reducing Fe^3+^ to Fe^2+^ from non-heme iron sources (Hacışevki, 2009). Four pathways are proposed for the biosynthesis of ascorbic acid in plants: D-mannose/L-galactose, galacturonate, myo-inositol, and gulose. However, the D-mannose/L-galactose pathway, for which initial precursor is D-glucose, is the only pathway regulated by light in non-genetically modified plants (Ntagkas et al., 2018). Recycling and turnover pathways also regulate ascorbic acid content (Zhang et al., 2011). In addition, to being the precursors for the biosynthesis of ascorbic acid, soluble carbohydrates also link respiration and photosynthesis. Moreover, the rate of photosynthesis regulates sucrose content and consequently glucose levels. However, the correlation between soluble carbohydrate content and ascorbic acid accumulation under light has not been established (Ntagkas et al., 2019).

β-carotene is the precursor of vitamin A and act as an antioxidant by scavenging free radicals and quenching singlet oxygen (Podsędek, 2007). From discussed metabolites, only β-carotene directly participates in light absorption, absorbing light in the blue region at 448 and 454 nm (peak absorption in acetone) (Lefsrud et al., 2008). Moreover, carotenoids are the primary determinant of photoprotective efficiency in plants (Cazzaniga et al., 2012).

The exposure of light spectrum (Brazaitytė et al., 2015), intensity (Kopsell et al., 2012), or dosage (Samuolienė et al., 2016; Samuolienė et al., 2017) are important factors affecting plant secondary metabolite production, in addition to other physiological changes. Notwithstanding, the nutrient composition of vegetables is very complex and challenging to assess. Recent studies show the potential for light-emitting diodes (LEDs) to regulate light quality and increase cellular metabolism and biosynthesis of defense-related secondary metabolites (Craver et al., 2017; Alrifai et al., 2019). Although microgreens solely with artificial lighting produce a continuous and uniform yield of high-quality products, but high-energy inputs are required. Artificial lighting is common in indoor short-vegetation cycle vegetable production, and recently, LED lamps have come into wide use in controlled-environment agriculture, including in plant factory and other indoor farms (Bantis et al., 2018). However, because of the use of artificial light, indoor plant production is one of the most energy-intensive forms of agriculture (Shamshiri et al., 2018). The successful development of new types of energy-saving, highly efficient luminous material technology can decrease the costs of LED light (Xu et al., 2016).

The important roles of far-red, red, blue, or ultraviolet photoreceptors (phytochromes, cryptochromes, phototropin, UVR8) in plant morphology and development and biosynthesis of phytochemicals are well described (Galvão and Fankhauser, 2015). However, the information on other supplementary components of light, particularly green LED light, is sparse. Because green light can penetrate further into leaf tissue, the responses of photosynthetic behavior and secondary metabolism to green light should receive greater attention.

Thus, the goal of this study was to investigate the effects of supplemental green, orange, and yellow LED light (supplemental components right above and close to the blue light spectrum) with basal red and blue lighting on the modulation secondary metabolism in microgreens of the *Brassicaceae*.

## Materials and Methods

### Growing Conditions and Lighting System

From the family Brassicaceae, the microgreens mizuna (*Brassica rapa ´Japonica´*), broccoli (*Brassica oleracea* ‘Green’), and kohlrabi (*Brassica oleracea* ‘Delicacy Purple’) (CN Seeds, Ltd., UK) were grown in peat substrate (Profi 1, Durpeta JSC, Lithuania) (pH 5–6) in 0.5 L plastic pot (18×11×6 cm) for 10 days from sowing to harvest. The nutrients concentrations in the substrate were N 110, P_2_O_5_ 50, K_2_O 160 (used as mg L^-1^); microelements (mg L^-1^)—Fe 4, Mn 0.2, Cu 0.1, B 2, Mo and Zn 0.1. Approximately 1.5 g of seeds was seeded per pot, which represented one replicate. Three pots were used under each lighting condition. Microgreens with cotyledons and stems harvested at ground level 10 days after germination. The samples were taken from the central part of the pot, plants were not sampled within 1.5 cm from the edge to avoid edge effect. The fresh tissue samples of microgreens were collected in plastic bags (6 × 8 cm) and stored in -80 °C in a freezer for biochemical analyses. Three biological replicates were analyzed for each of the biochemical analyses. The experiments were performed in controlled-environment growth chambers. The day/night temperatures were +21/17 ± 2°C, the photoperiod was 16 h, and the relative air humidity of 50%–60%. The four LED lighting treatments were blue-red (BR), blue-red-green (BRG), blue-red yellow (BRY), and blue-red-orange (BRO) (**Table 1**) by Tamulaitis et al. (2005) originally designed the LED-based lighting units, which consisted of LED emission wavelengths of blue (LXHL–LR3C, 447 nm), red (LXHL-LD3C, 638 nm and LXM3-PD01-0300, 665 nm) (Philips Lumileds, USA), far-red (L731-05-AU, 731 nm) (Epitex, Japan), green (LXHL-MM1D, 520 nm), yellow (LXHL-MLAC, 595 nm), and orange (LXHL-MLAC, 622 nm). The surface area under the lighting unit was approximately 0.5 m^2^. The photosynthetic photon flux density (PPFD) was set at 300 µmol m^-2^ s^-1^. The PPFD was selected as the optimal lighting intensity on the basis of previous experiments (Samuolienė et al., 2013). The PPFD was measured using a photometer–radiometer (RF-100, Sonopan, Poland) at the top of a pot, not less than five points under the illuminated area, including the center and periphery. The average PPFD value was set at 300 µmol m^-2^ s^-1^.

**Table 1:** The wavelengths and photosynthetic photon flux densities (PPFD’s) of the applied LED spectra.

Treatment	Blue, 447 nm	Red, 638 nm	Red, 665 nm	Far-red, 731 nm	Green, 520 nm	Yellow, 595 nm	Orange, 622 nm
PPFD, µmol m^-2^ s^-1^							
BR	42	104	150	4			
BRG	42	89	150	4	15		
BRY	42	89	150	4		15	
BRO	42	89	150	4			15

### Determination of Dry Weight

The dry weight (DW) of 20 randomly selected plants from each experimental replication per treatment was determined. The fresh plants were dried at 105°C for 24 h (Venticell 222, MBT, the Czech Republic) to constant weight (Mettler-Toledo AG64, USA). The contents of analyzed metabolites and elements were calculated on a DW basis.

### Determination of Sugars

Approximately 0.5 g of fresh plant tissue was ground and diluted with deionized H_2_O. The extraction was conducted for 4 h at room temperature with mixing. The samples were then centrifuged at 14,000× g for 15 min. A cleanup step was performed before the chromatographic analysis. Briefly, 1 ml of supernatant was mixed with 1 ml of 0.01% (w/v) ammonium acetate in acetonitrile and incubated for 30 min at +4°C. The samples were centrifuged at 14,000× g for 15 min and filtered through a 0.22 µm PTPE syringe filter (VWR International, United States). The analyses were performed on a Shimadzu HPLC (Japan) instrument equipped with an evaporative light scattering detector (ELSD). The separation of fructose, glucose, and sucrose was performed on a Shodex VG-50 4D HPLC column with a deionized water (mobile phase A) and acetonitrile (mobile phase B) gradient. The gradient was maintained at 88% B for 13 min, changed linearly to 70% B in 9 min, kept at 70% B for 1 min, raised back to 88% B in 2 min and the column was equilibrated to 88% B for 5 min. The flow rate was 0.8 ml min^-1^.

### Determination of Ascorbic Acid

The ascorbic acid content was evaluated using a spectrophotometric method (Janghel et al., 2007). One gram of fresh plant tissue was homogenized in 10 ml of 5% oxalic acid in order to avoid the loss of ascorbic acid and then centrifuged (5 min, 4000 rpm min^-1^). The extract 1 ml was mixed with 2 ml of 0.1% methyl viologen and 2 ml 2 mol L^-1^ sodium hydroxide. The solution was shaken gently and allowed to stand for 2 min. The colored radical ion was measured at 600 nm against the radical blank.

### Determination of β-Carotene

β-carotene was extracted using 80% acetone (1 g of sample ground with liquid N and 10 ml^-1^ of solvent) and then centrifuged (5 min, 4000 rpm min^-1^) and filtered through a 0.45-µm nylon membrane syringe filter (VWR International, United States). The contents of β-carotene were evaluated using a Shimadzu HPLC (Japan) instrument equipped with a diode array detector (SPD-M 10A VP) on a YMC Carotenoid column (3 µm particle size, 150 × 4.0 mm) (YMC, Japan). The mobile phase consisted of A (80% methanol, 20% water) and B (100% ethyl acetate). The gradient was as follows: 0 min; 20% B, 2.5 min; 22.5% B, 20–22.5 min; 50% B, 24-26 min; 80% B, 31–34 min; 100% B, 42–47 min; and 20% B, flow rate 1 ml min^-1^ (Edelenbos et al., 2001). The peak was detected at 440 nm and identified using an external calibration method.

### Determination of Mineral Elements

The contents of the macroelements calcium (Ca), potassium (K), and magnesium (Mg) in microgreens were determined by using a modified microwave-assisted digestion technique combined with inductively coupled plasma optical emission spectrometry (ICP-OES) (Araújo et al., 2002; Barbosa et al., 2015). The complete digestion of 0.5 g of dry matter was achieved with 65% nitric acid (HNO_3_) and 30% hydrogen peroxide (H_2_O_2_) (5:3) using the microwave digestion system Multiwave GO (Anton Paar GmbH, Austria). A two-step program was selected with a maximum temperature of 180°C: 1) 150°C was reached within 3 min, followed by digestion for 10 min; and 2) 180°C was reached within 10 min, followed by digestion for 10 min. The mineralized samples were diluted to 50 ml with ultrapure water (Purelab Flex, Elga, United Kingdom). The elemental profile was analyzed with an ICP–OES SPECTRO Genesis spectrometer (Spectro Analytical Instruments GmbH, Germany). The contents of Mg, Ca, and K (mg L^-1^) were evaluated at the analytical wavelengths of 279.079, 445.478, and 766.491 nm, respectively, using the ICP-OES multi-elemental standard solution (Merck KGaA, Germany), in the range of 0.01–400 mg L^-1^. Each mineral analysis was performed with three analytical replications.

### Statistical Analyses

The data were processed using XLStat software (Addinsoft, 2019, United States), and analysed using one-way analysis of variance (ANOVA), followed by Duncan’s multiple range test at the confidence level p = 0.05. Data was processed using MS Excel software (version 7.0), standard deviation represents the mean of three replicates and is expressed on a DW basis.Multivariate principal component analysis (PCA) was performed: The results are presented in PCA scatter plot that indicate distinct metabolisms and levels of mineral nutrition in microgreens under the different lighting spectra and a correlation circle (based on Pearson’s correlation matrix) that summarizes the metabolic relations between investigated metabolites and minerals under the different lighting spectra.

## Results

**Figure 1:**
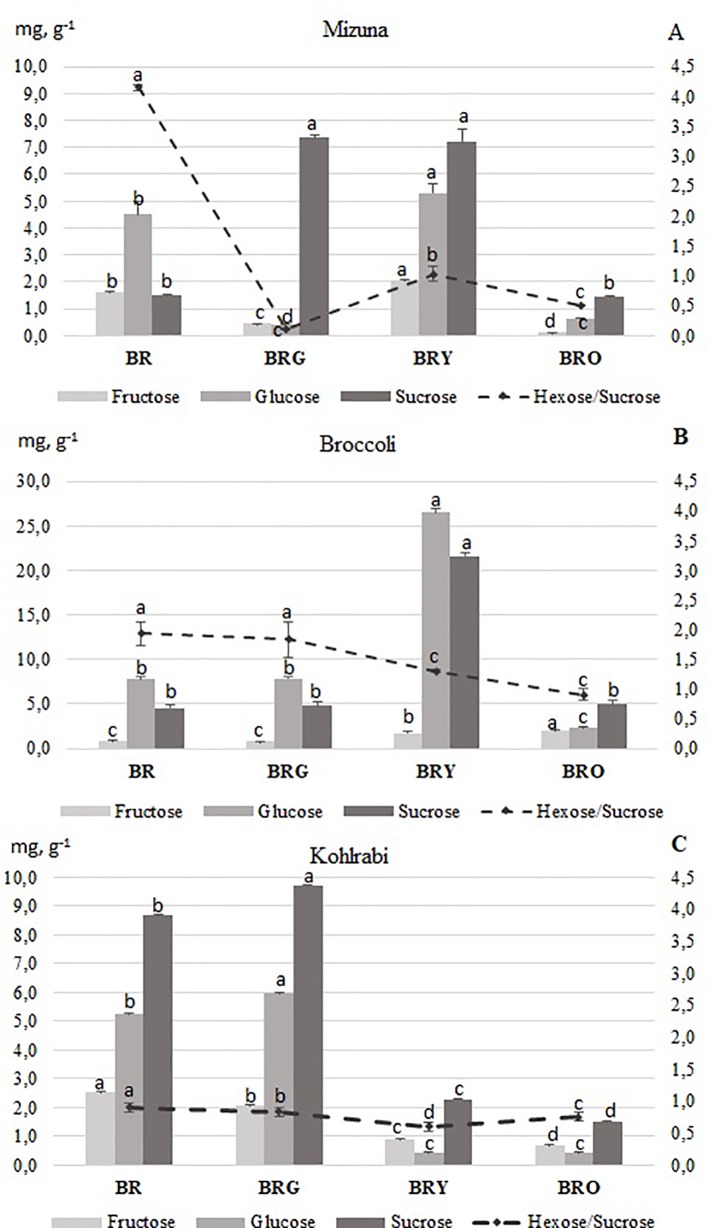
Soluble sugar contents in mizuna **(A)**, broccoli **(B)** and kohlrabi **(C)** microgreens. BR—blue (447 nm), red (638 and 665 nm), far-red (731 nm); BRG—BR with supplemental green (520 nm); BRY—BR with supplemental yellow (595 nm); BRO—BR with supplemental orange (622 nm). Total PPFD maintained at 300 µmol m^-1^ s^-2^, changing the input of red 665 nm. The data were processed using one-way analysis of variance (ANOVA), Duncan’s multiple range test at the confidence level p = 0.05. Presented values marked by similar letters do not differ significantly. Data was processed using MS Excel software (version 7.0), standard deviation represents the mean of three replicates and is expressed on a DW basis. Samples were taken from the central vessel part, and one vessel represented one replicate, three biological replicates were performed (n = 3). BR = blue-red, BRG = blue-red-green, BRY = blue-red yellow, and BRO = blue-red-orange.

In the control (BR) treatment, the highest total sugar content was: kohlrabi (16.44 mg g^-1^, DW), followed by broccoli (13.06 mg g^-1^, DW) and mizuna (7.59mg g^-1^, DW) (**Figure 1**). In the supplemental BRG and BRY light treatments the content of sucrose increased significantly (5-fold), and in the BRY treatment the contents of hexoses also increased significantly in mizuna (**Figure 1A**). Compared with the other lighting treatments, the glucose and sucrose increased significantly (4.5-fold) in broccoli under the BRY treatment (**Figure 1B**). In addition, the of fructose (2.9-fold), glucose (13.0-fold), and sucrose (4.9-fold) in broccoli increased significantly under the BR and BRG treatments, compared with those under supplemental yellow (BRY) or orange (BRO) light (**Figure 1C**). The hexoses to sucrose ratio was the highest significantly, primarily due to high glucose contents, under the BR and BRG treatments for all microgreens, except for mizuna under BRG light (**Figure 1**).

In the control (BR) treatment, the highest ascorbic acid content was: broccoli (106.4 mg g^-1^, DW), followed by kohlrabi (35.0 mg g^-1^, DW) and mizuna (21.1 mg g^-1^, DW) (**Table 2**). By contrast, such drastic differences were not observed in β-carotene contents in treated microgreens under the BR treatment, except for a decrease in β-carotene (33.4%) in kohlrabi. A common response was not observed for ascorbic acid accumulation, with a significant increases in mizuna (3.0-fold) under the BRO treatment, in broccoli (from 1.2 to 4.2-fold) under the BRG treatment, and in kohlrabi (1.3-fold) under BRY treatment. However, compared with BRO, significant decrease occurred in ascorbic acid content in mizuna [3.5-fold and in broccoli (3.6-fold) under BRY]. In contrast to mizuna, the content of β-carotene under the BR treatment increased significantly in broccoli (1.7-fold compared with that under BRG and BRO and 58-fold compared with that under BRY) and in kohlrabi (1.5-fold compared with that under BRG and 5.2-fold compared with that under BRY and BRO). However, under the BRY treatment the β-carotene content increased significantly in mizuna but decreased significantly in broccoli (45.7-fold) and in kohlrabi (4.6-fold) (**Table 2**).

**Table 2:** Variation of ascorbic acid (AscA, mg g^-1^ DW) and β-carotene (β-Car, mg g^-1^ DW) in mizuna, broccoli and kohlrabi microgreens.

Treatment	Mizuna		Kohlrabi		Kohlrabi	
	AscA	β-Car	AscA	β-Car	AscA	β-Car
BR	21.1^b^	0.57^c^	106.4^b^	0.58^a^	35.0^d^	0.44^a^
BRG	20.2^bc^	1.26^a^	123.0^a^	0.39^b^	49.9^b^	0.30^b^
BRY	16.7^c^	1.17^a^	29.1^d^	0.01^c^	54.3^a^	0.08^c^
BRO	57.7^a^	1.00^b^	85.4^c^	0.40^b^	44.0^c^	0.09^c^

BR—blue (447 nm), red (638 and 665 nm), far-red (731 nm); BRG—BR with supplemental green (520 nm); BRY—BR with supplemental yellow (595 nm); BRO—BR with supplemental orange (622 nm). Total PPFD maintained at 300 µmol m^-2^ s^-1^, changing the input of red 665 nm. The data were processed using one-way analysis of variance (ANOVA), Duncan’s multiple range test at the confidence level p = 0.05. Presented values marked by similar letters do not differ significantly. Samples were taken from the central vessel part, and one vessel represented one replicate, three biological replicates were performed (n = 3). BR, blue-red; BRG, blue-red-green; BRY, blue-red yellow; and BRO, blue-red-orange.

In the control (BR) treatment, the accumulation of Fe, Mg, and Ca was not significantly different among microgreens (**Table 3**). Only the BRO treatment resulted in significant increase in mineral element contents in mizuna (1.3-fold), broccoli (2.0-fold), 1.9-fold for Fe, and 1.5 times for Mg and Ca in kohlrabi.

**Table 3:** Micro (Fe, mg g^-1^ DW) and macro (Mg, Ca, mg g^-1^ DW) elements contents in mizuna, broccoli and kohlrabi microgreens.

Treatment	Mizuna			Broccoli			Kohlrabi		
	Fe	Mg	Ca	Fe	Mg	Ca	Fe	Mg	Ca
BR	0.32^c^	4.39^b^	17.9^b^	0.36^c^	4.87^b^	15.5^b^	0.28^b^	5.04^b^	18.8^b^
BRG	0.30^c^	4.19^b^	17.0^b^	0.35^c^	4.54^b^	14.3^b^	0.32^b^	5.48^b^	21.0^b^
BRY	0.43^b^	3.80^c^	15.2^c^	0.43^b^	5.08^b^	16.6^b^	0.38^b^	5.22^b^	20.0^b^
BRO	0.49^a^	5.16^a^	21.7^a^	0.84^a^	9.76^a^	31.0^a^	0.62^a^	7.65^a^	29.9^a^

BR—blue (447 nm), red (638 and 665 nm), far-red (731 nm); BRG—BR with supplemental green (520 nm); BRY—BR with supplemental yellow (595 nm); BRO—BR with supplemental orange (622 nm). Total PPFD maintained at 300 µmol m^-2^ s^-1^, changing the input of red 665 nm. The data were processed using one-way analysis of variance (ANOVA), Duncan’s multiple range test at the confidence level p = 0.05. Presented values marked by similar letters do not differ significantly. Samples were taken from the central vessel part, and one vessel represented one replicate, three biological replicates were performed (n = 3). BR, blue-red; BRG, blue-red-green; BRY, blue-red yellow; and BRO, blue-red-orange.

**Figure 2:**
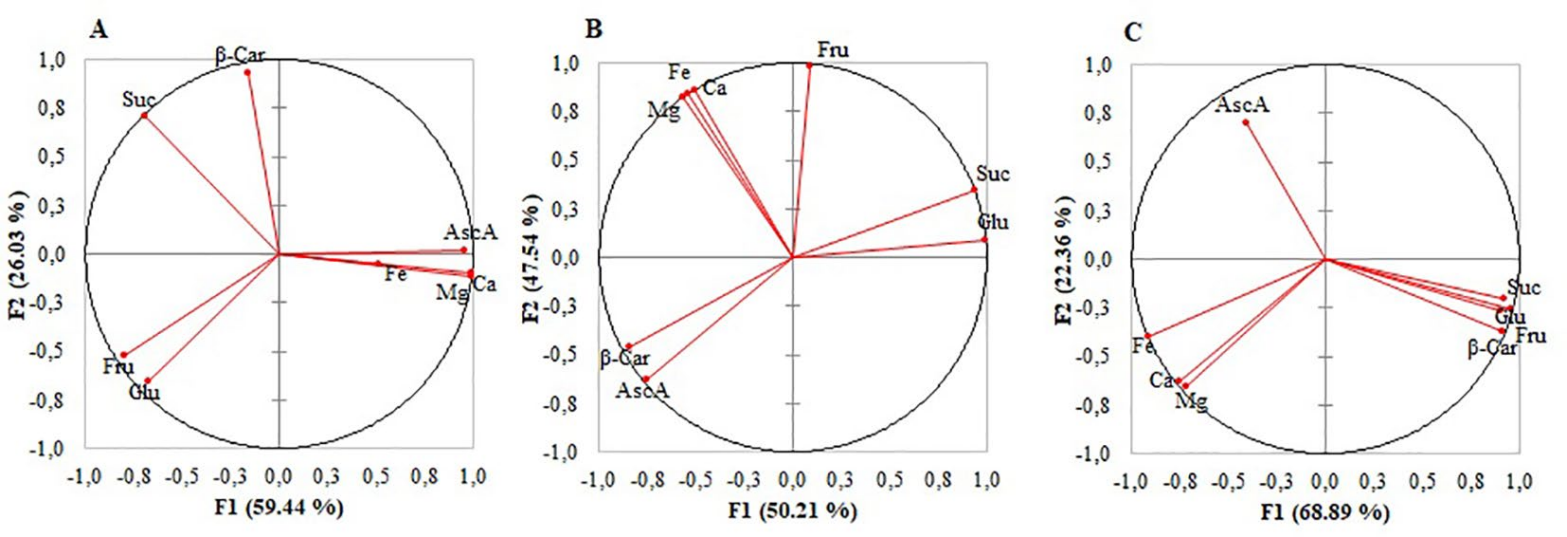
Correlation circle of metabolites and mineral elements in mizuna **(A)**, broccoli **(B)**, and kohlrabi **(C)** microgreens.

The correlation circles of metabolites and mineral elements showed the results were uneven among the microgreens (**Figure 2**). A very strong or strong negative correlation between ascorbic acid and sugars was found in mizuna (**Figure 2A** and **Table S1**) and broccoli (**Figure 2B**, **Table S1**), whereas in kohlrabi (**Figure 2C** and **Table S1**) the correlation was moderate or weak. In broccoli ascorbic acid and β-carotene were strongly positively correlated, whereas in kohlrabi, the correlation was strongly negative. Ca, Mg, and sugars were strongly or moderately negatively correlated in mizuna and kohlrabi, respectively. Whereas only in kohlrabi Fe, sugars, and β-carotene were strongly negatively correlated. A strong positive correlation was observed between Fe and ascorbic acid in mizuna and between Fe and fructose in broccoli. A very strong positive correlation was observed between Fe and Ca and between Fe and Mg in broccoli and kohlrabi, whereas in mizuna, those correlations were moderate. The contents of Ca and Mg very strongly positively correlated in all microgreens (**Table S1**).

**Figure 3:**
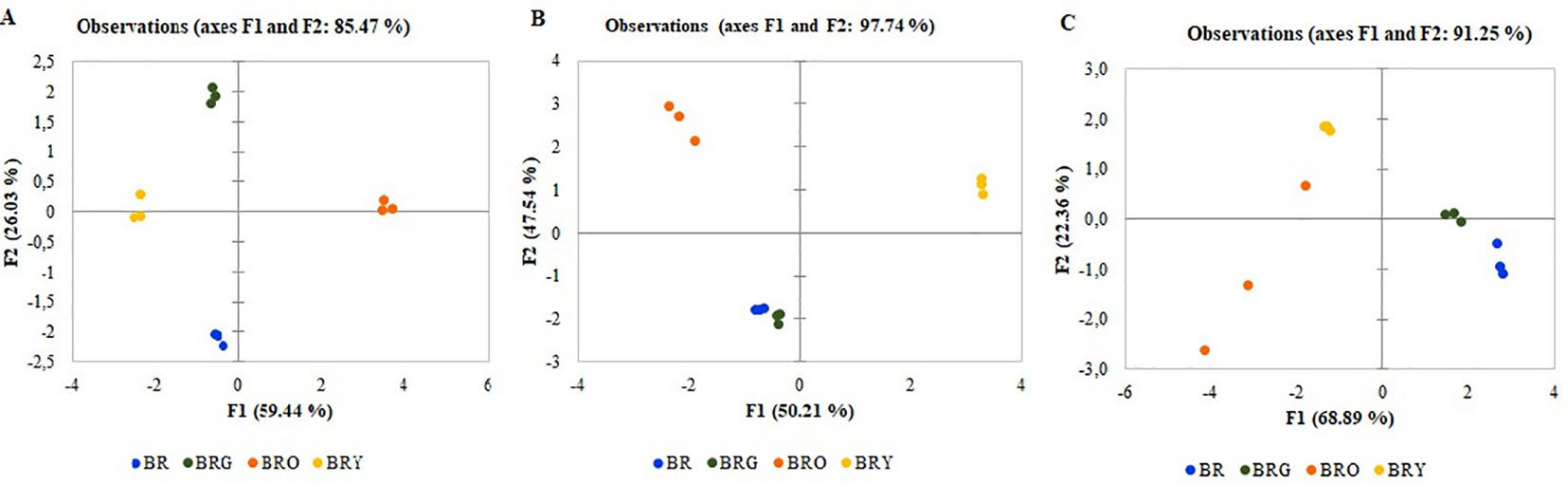
The PCA scatterplot, indicating distinct differences in metabolites and mineral elements in mizuna **(A)**, broccoli **(B)**, and kohlrabi **(C)** microgreens. BR—blue (447 nm), red (638 and 665 nm), far-red (731 nm); BRG—BR with supplemental green (520 nm); BRY—BR with supplemental yellow (595 nm); BRO—BR with supplemental orange (622 nm). Total PPFD maintained at 300 µmol m^-2^ s^-1^, changing the input of red 665 nm.

The results of the PCA show the average coordinates of individual sugars (sucrose, fructose, glucose), mineral nutrients (Fe, Mg, Ca), β-carotene, and ascorbic acid under supplemental green, orange, and yellow lighting. The first two factors (F1 vs. F2) of the PCA, as shown in the correlation circle (**Figure 2**) and scatterplot (**Figure 3**), explained 97.74% of the total data variance of broccoli, 91.25% for that of kohlrabi, and 85.47% for that of mizuna. The F1 explained 50.21% (broccoli), 68%, 89% (kohlrabi), and 59%, 44% (mizuna) of the total variance, whereas F2 explained 47.57% (broccoli), 22.36% (kohlrabi), and 26.03% (mizuna) of the total variability. Thus, F1 described the disparity among supplemental lighting treatments. To summarize all effects in the PCA scatter plot, the common reaction of all microgreens to the BRG treatment was not significantly different from that under the BR treatment (**Figure 3**).However, the reaction of all microgreens to the BRY treatment was significantly different from that under BRG and BR treatment. The most distinct response was to the BRO treatment: the response was not different from that to BRY in mizuna (**Figure 3A**), was different from that to BRY in broccoli (**Figure 3B**), and was not significantly different in kohlrabi (**Figure 3C**) due to significant BRO scattering. However, the reaction to BRO was significantly different from that to BR in all microgreens (**Figure S1**).

## Discussion

Light spectral quality has a pronounced but differential effect on the accumulation of secondary plant metabolites in vegetables, including microgreens, cultivated in closed-environment facilities (Brazaitytė et al., 2015; Bantis et al., 2018; Alrifai et al., 2019). The differences in the quality of spectral light under far-red, red, and blue light or their combinations, as well as close-wavelength supplemental spectrum components, can variably affect the accumulation of bioactive compounds, with the effect also likely dependent on the plant species (Ohashi-Kaneko et al., 2007; Samuolienė et al., 2016). That the biosynthesis and accumulation of secondary metabolites are likely more pronounced under monochromatic blue or red LED light, when compared with conventional white light (Li and Kubota, 2009; Johkan et al., 2010). Because the red wavebands match the absorption peaks of chlorophylls and phytochrome receptors (Galvão and Fankhauser, 2015) red light would be the most efficient in supplementing existing light conditions to aid photosynthesis and further stimulate the biosynthesis of soluble sugars. By contrast, blue light acts *via* cryptochromes and stimulates the accumulation of carotenoids (Lefsrud et al., 2008). Kopsell and Sams (2013) found that compared with red LEDs, short-duration high PPFD blue light increased the content of β-carotene, among other metabolites, and essential nutrients, including Fe, Mg, and Ca. However, an inhibitory effect on iron absorption may be associated not only with the effect of light but also with the action of phytate, oxalate, or some polyphenols (Hacışevki, 2009; Pehlivan, 2017). The combination of red and blue LED lights increased chlorophyll, carotenoid, ascorbic acid, and soluble sugar contents in leaf lettuce, ascorbic acid and soluble sugars in spinach, and only soluble sugars in komatsuna, compared with single red, blue, or white LEDs (Ohashi-Kaneko et al., 2007). In this study, under the BR treatment, soluble sugars increased only in kohlrabi, whereas significant accumulations of glucose and sucrose in kohlrabi, and sucrose in mizuna occurred under supplemental green light. Under supplemental yellow light soluble sugars accumulated in mizuna and broccoli. However, in all microgreens the hexoses to sucrose ratio increased significantly only in BR treatment. Glucose is an initial precursor of ascorbic acid biosynthesis *via* the D-mannose/L-galactose pathway (Ntagkas et al., 2018) and regulates the light-dependent reactions of ascorbic acid (Ntagkas et al., 2019). However, the correlations between glucose and ascorbic acid were negative, with the correlation weak in kohlrabi, moderate in mizuna, and very strong in broccoli. Thus, the glucose substrate may be effectively exploited for ascorbic acid biosynthesis through the D-mannose/L-galactose pathway. Ntagkas et al. (2019) found that the light-induced accumulation of ascorbic acid is independent of the carbohydrate content in tomato fruits. The negative correlation between Mg and sucrose in all the microgreens might be related to Mg regulation of sucrose loading into phloem or carbon accumulation in source leaves. According to Cakmak and Kirkby (2008), Mg acts as an enzyme activator or cofactor in carbohydrate metabolism, and thus Mg deficiency inhibits enzyme activity and leads to further carbon accumulation in source leaves. Up to 20% of total Mg is associated with chlorophyll pigments and acts as a cofactor in a series of enzymes involved in photosynthetic carbon fixation and metabolism (Hermans et al., 2013). However, the content of Mg, as well as that of Fe and Ca, was the highest significantly under supplemental orange light in all microgreens, whereas BR and BRG treatments suppressed the accumulation of Mg. Furthermore, some blue and green light-reversible effects are blue to green light ratio-dependent, in addition to green light acting antagonistically to blue light by inactivating blue light responses (Smith et al., 2017). However, the common reaction of the *Brassicacea* microgreens to supplemental green light was not significantly different from that BR light. Dougher and Bugbee (2001) found that short- and long-wavelength green light (500–600 nm) cause different responses within a plant. Thus, these results demonstrate the unequal action of green light is caused by different wavelengths. Moreover, in studies based on increasing doses of yellow light (580–600 nm), the yield of lettuce decreases (Dougher and Bugbee, 2001). In this study, the reaction of all microgreens to supplemental yellow light was significantly different from that to BRG and BR light. Thus, the application of the appropriate wavelengths and intensities of green may benefit secondary metabolism. Fe, Mg, and Ca were very strongly positively correlated in all microgreens, except in mizuna, in which a moderately positive correlation was observed between Fe and Ca and between Fe and Mg. Guo et al. (2016) suggest that plant cells compensate for low Ca by increasing Mg transporter activity, whereas high Ca concentration inhibits Mg^2+^ availability to plants. The interaction between ions is also an important factor, because the accumulation of Fe is negatively associated with concentrations of Ca, Mg, and Mn in alfalfa, broccoli, and radish sprouts (Park et al., 2014). In studies on microgreens, supplemental green light increases the contents of α-carotene and β-carotene (Kopsell et al., 2014; Brazaitytė et al., 2015; Brazaitytė et al., 2016; Samuolienė et al., 2017). However, supplemental orange LEDs lead to decreases in β-carotene content (Brazaitytė et al., 2015). Notwithstanding, β-carotene directly participates in light absorption, absorbing light in the blue region (Lefsrud et al., 2008). In this study, β-carotene increased significantly in mizuna under supplemental green and yellow light and in broccoli and kohlrabi under BR light.

## Conclusions

The promoters and inhibitors of Fe absorption strongly regulated Fe accumulation. A very strong positive correlation was observed between Fe and Ca and between Fe and Mg in kohlrabi and broccoli, whereas, a strong negative correlation was observed between Fe and β-carotene and between Fe and soluble carbohydrates only in kohlrabi. In contrast to the supplemental yellow light, the common reaction of tested microgreens to supplemental green light was not significantly different from that with BR light. Thus, the metabolic changes that occurred in treated microgreens led to increases in the contents of essential nutrients. Therefore, to preserve and increase the contents of certain nutritionally valuable metabolites, selected supplemental LED wavelengths might benefit the cultivation of *Brassicaceae* microgreens.

## Data Availability Statement

The datasets generated for this study are available on request to the corresponding author.

## Author Contributions

GS—data analysis, spectrophotometric analysis, writing of the manuscript. AB, PD—joint coordination of the experiment, modeling of light parameters, data summarizing. AV—the realization of lighting schedules in vegetative experiments, data analysis. JM—microwave digestion of mineral elements combined with ICP-OES. VV-K—chromatographic analysis. All authors read and approved the final version of the manuscript.

## Funding

This research was funded by a Grant (No. SVE-03/2011) from the Research Council of Lithuania.

## Conflict of Interest

The authors declare that the research was conducted in the absence of any commercial or financial relationships that could be construed as a potential conflict of interest.
