# Supplementary figures and images for: Nutrient Levels in Brassicaceae Microgreens Increase Under Tailored Light-Emitting Diode Spectra
Source: Front Plant Sci. 2019 Nov 14;10:1475. doi: 10.3389/fpls.2019.01475 (PMC6868063; doi:10.3389/fpls.2019.01475)

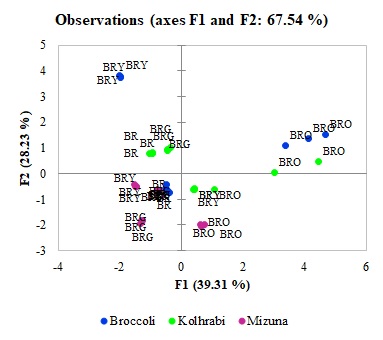

Supplement: Supplementary file 1 [file Image_1.jpg]
