# Supplementary material for: Nutrient Levels in Brassicaceae Microgreens Increase Under Tailored Light-Emitting Diode Spectra
Source: Front Plant Sci. 2019 Nov 14;10:1475. doi: 10.3389/fpls.2019.01475 (PMC6868063; doi:10.3389/fpls.2019.01475)
Supplement: Supplementary file 2 [file Table_1.docx]

**SUPPLEMENTRY TABLE**

**Table S1** The correlation matrix (Pearson (n)) between metabolites and mineral elements in mizuna, broccoli and kohlrabi microgreens.

| **Variables** | | | **AscA** | | | **Fructose** | | | **Glucose** | | | **Sucrose** | | | **β-Car** | | | **Fe** | | | **Ca** | | | **Mg** | | |
| --- | --- | --- | --- | --- | --- | --- | --- | --- | --- | --- | --- | --- | --- | --- | --- | --- | --- | --- | --- | --- | --- | --- | --- | --- | --- | --- |
| **Mizuna** | | | | | | | | | | | | | | | | | | | | | | | | | | |
| AscA | | | **1** | | |  | | |  | | |  | | |  | | |  | | |  | | |  | | |
| Fructose | | | **-0.710** | | | **1** | | |  | | |  | | |  | | |  | | |  | | |  | | |
| Glucose | | | **-0.579** | | | **0.964** | | | **1** | | |  | | |  | | |  | | |  | | |  | | |
| Sucrose | | | **-0.623** | | | **0.222** | | | **0.050** | | | **1** | | |  | | |  | | |  | | |  | | |
| β-Car | | | **-0.055** | | | **-0.262** | | | **-0.387** | | | **0.802** | | | **1** | | |  | | |  | | |  | | |
| Fe | | | **0.705** | | | **-0.151** | | | **-0.022** | | | **-0.282** | | | **0.148** | | | **1** | | |  | | |  | | |
| Ca | | | **0.936** | | | **-0.758** | | | **-0.616** | | | **-0.763** | | | **-0.262** | | | **0.476** | | | **1** | | |  | | |
| Mg | | | **0.929** | | | **-0.750** | | | **-0.605** | | | **-0.777** | | | **-0.285** | | | **0.463** | | | **0.999** | | | **1** | | |
| **Broccoli** | | | | | | | | | | | | | | | | | | | | | | | | | | |
| AscA | | **1** | | |  | | |  | | |  | | |  | | |  | | |  | | |  | | |  |
| Fructose | | **-0.686** | | | **1** | | |  | | |  | | |  | | |  | | |  | | |  | | |  |
| Glucose | | **-0.815** | | | **0.170** | | | **1** | | |  | | |  | | |  | | |  | | |  | | |  |
| Sucrose | | **-0.926** | | | **0.408** | | | **0.965** | | | **1** | | |  | | |  | | |  | | |  | | |  |
| β-Car | | **0.866** | | | **-0.509** | | | **-0.863** | | | **-0.935** | | | **1** | | |  | | |  | | |  | | |  |
| Fe | | **-0.151** | | | **0.787** | | | **-0.427** | | | **-0.180** | | | **0.024** | | | **1** | | |  | | |  | | |  |
| Ca | | **-0.120** | | | **0.761** | | | **-0.458** | | | **-0.215** | | | **0.068** | | | **0.998** | | | **1** | | |  | | |  |
| Mg | | **-0.087** | | | **0.741** | | | **-0.486** | | | **-0.247** | | | **0.094** | | | **0.996** | | | **0.999** | | | **1** | | |  |
| **Kohlrabi** | | | | | | | | | | | | | | | | | | | | | | | | | |  |
| AscA | **1** | | |  | | |  | | |  | | |  | | |  | | |  | | |  | | |  |  |
| Fructose | **-0.546** | | | **1** | | |  | | |  | | |  | | |  | | |  | | |  | | |  |  |
| Glucose | **-0.383** | | | **0.946** | | | **1** | | |  | | |  | | |  | | |  | | |  | | |  |  |
| Sucrose | **-0.346** | | | **0.951** | | | **0.997** | | | **1** | | |  | | |  | | |  | | |  | | |  |  |
| β-Car | **-0.670** | | | **0.969** | | | **0.890** | | | **0.886** | | | **1** | | |  | | |  | | |  | | |  |  |
| Fe | **0.104** | | | **-0.783** | | | **-0.709** | | | **-0.751** | | | **-0.687** | | | **1** | | |  | | |  | | |  |  |
| Ca | -0.012 | | | **-0.553** | | | **-0.455** | | | **-0.502** | | | **-0.466** | | | **0.931** | | | **1** | | |  | | |  |  |
| Mg | **-0.042** | | | **-0.509** | | | **-0.420** | | | **-0.466** | | | **-0.421** | | | **0.907** | | | **0.997** | | | **1** | | |  |  |

Values in bold are different from 0 with a significance level α=0.95
